# Successful Mechanical Thrombectomy for Acute Middle Cerebral Artery Occlusion in a Centenarian

**DOI:** 10.7759/cureus.22071

**Published:** 2022-02-09

**Authors:** Hiroyasu Inoue, Masahiro Oomura, Yusuke Nishikawa, Mitsuhito Mase, Noriyuki Matsukawa

**Affiliations:** 1 Neurology, Nagoya City University Graduate School of Medical Sciences, Nagoya, JPN; 2 Neurosurgery, Nagoya City University Graduate School of Medical Sciences, Nagoya, JPN

**Keywords:** large vessel occlusions, acute ischemic stroke, mechanical thrombectomy, super elderly, oldest, centenarian

## Abstract

Clinical trials have proven the efficacy and beneficial therapeutic outcomes of endovascular therapy in patients with major arterial occlusion. However, its efficacy for very elderly patients, such as nonagenarians or centenarians, is not well established. In this case report, we describe the successful use of mechanical thrombectomy for the management of stroke in a centenarian. The 100-year-old woman with severe right-sided paralysis and total aphasia was admitted to our hospital approximately 30 min from the onset of symptoms. The National Institutes of Health Stroke Scale score was 24 at admission, and three-dimensional computed tomography angiography revealed occlusion in the M1 segment of left middle cerebral artery. She had persistent atrial fibrillation and was diagnosed with colon cancer one month prior to the admission. The modified Rankin Scale score before the stroke was 1, and she was generally independent. The patient successfully underwent mechanical thrombectomy, and recanalization with thrombolysis in cerebral infarction grade 3 was accomplished 129 minutes after the onset. The patient made a remarkable recovery with a National Institutes of Health Stroke Scale score of 4 at 48 h and was discharged home with a modified Rankin Scale score of 2 on day 8. Thus, mechanical thrombectomy can be performed with a good outcome even in centenarians.

## Introduction

Although clinical trials have proven the efficacy and beneficial therapeutic outcomes of endovascular therapy in patients with large-vessel occlusion (LVO) [1-5], its efficacy in very elderly patients, such as nonagenarians or centenarians, is yet to be established. A study reported that mechanical thrombectomy (MT) does not lead to good outcomes in patients over 80 years of age [6], whereas another stated that MT can improve outcomes in patients over 90 years of age [7]. There are even fewer reports in the literature on centenarians [8-11]. Herein, we describe the case of a 100-year-old woman who underwent MT and had a good outcome.

## Case presentation

**Figure 1:**
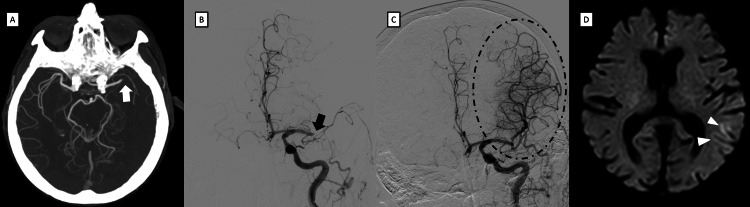
Images of our patient from admission to two days after MT. (A) Computed tomography angiography showing occlusion of the M1 segment of the left middle cerebral artery (white arrow). (B) Cerebral angiography showing distal occlusion of the M1 segment of the left middle cerebral artery (black arrow). (C) Complete recanalization was achieved with the first-pass effect using the combined technique (dot circle). (D) Magnetic resonance diffusion-weighted imaging two days later showed only slight infarcts in the left temporal lobe (white arrowheads). MT: mechanical thrombectomy.

A 100-year-old female developed sudden onset severe right-sided paralysis and aphasia in the presence of her daughter at 15:00. She had persistent atrial fibrillation and was diagnosed with colon cancer a month earlier. She had no diabetes or dyslipidemia. She lived alone, and prior to the stroke, she was able to independently perform toileting and bathing and was generally independent in daily activities, with a modified Rankin Scale (mRS) score of 1. She was admitted to the hospital at 15:31 with a National Institutes of Health Stroke Scale (NIHSS) score of 24. Three-dimensional computed tomography (CT) angiography at 15:50 revealed occlusion of the M1 segment of the left middle cerebral artery with an Alberta Stroke Program Early CT Score of 10 (Figure 1A). She was treated with direct endovascular therapy without recombinant tissue plasminogen activator because of a recent history of bloody stool. Groin puncture was performed at 16:32, and a FlowGate Balloon Guide Catheter (8 Fr × 85 cm; Stryker Neurovascular, Fremont, CA, USA) was placed in the left internal carotid artery (ICA). A Trevo 4 × 20 mm stent retriever (Stryker Neurovascular) was deployed from M2 to M1 while controlling blood flow on the proximal side. A Catalyst 6 aspiration catheter (Stryker Neurovascular) was placed in contact with the Trevo and removed together while applying aspiration pressure. A red thrombus was removed, and complete recanalization with thrombolysis in cerebral infarction grade 3 was achieved with the first-pass effect at 17:09, 129 min after symptom onset (Figures 1B, 1C). Neurological deficits improved immediately after treatment, with NIHSS scores of 8 and 4 on postintervention days 1 and 2, respectively. Magnetic resonance diffusion-weighted imaging performed two days later showed only a very small infarct (Figure 1D). Although slight motor aphasia remained, she was discharged home after eight days of hospitalization, with an mRS of 2 (Videos 1, 2).

**Video 1:** The patient walks around the hospital room on the fifth day of treatment.

**Video 2:** The patient eats with chopsticks in her right hand on the fifth day of treatment.

## Discussion

In Japan, the number of people aged ≥90 years is increasing every year, with more than 2.5 million people aged ≥90 years and more than 80,000 aged ≥100 years reported in 2021 [12]. The incidence of cerebrovascular diseases, such as subarachnoid hemorrhage (SAH), among the elderly is increasing along with the aging of the population [13]. Additionally, because the incidence of atrial fibrillation and the proportion of cardiogenic cerebral emboli leading to cerebral infarctions increase with age [14], the number of LVOs in the very elderly is expected to increase in the future.

Based on its established efficacy, MT immediately after LVO onset has obtained class 1 recommendation in the American Heart Association (AHA)/American Stroke Association (ASA) guidelines, and the selection criteria do not have an upper age limit [15]. However, two of the five randomized controlled trials (RCTs) that established the efficacy of MT excluded patients aged >80 or >85 years, respectively [1,2]. In the remaining three, only a minority of patients were aged >80 years and even fewer were aged >90 years [3-5]. Thus, the efficacy of MT in very old patients remains unclear. Recently, one study showed that even in patients aged >90 years, MT improves outcomes [7]. However, poor outcomes with MT have also been reported in patients >90 years of age [16]. Besides our case, there have been four reports of MT in centenarians (Table 1) [8-11].

**Table 1:** Mechanical thrombectomy in centenarians. mRS, modified Rankin Scale; NIHSS, National Institutes of Health Stroke Scale; ASPECTS, Alberta Stroke Program Early Computed Tomography Score; rt-PA, recombinant tissue plasminogen activator; TICI, thrombolysis in cerebral infarction; N/A, not available; CES, cardioembolic stroke; F, female; ICA-T, internal carotid artery terminus; ICA, internal carotid artery; ATBI, atherothrombotic brain infarction; M, male.

Report details	Age	Sex	Preadmission mRS	NIHSS at onset	ASPECTS	rt-PA	Occluded vessel	Etiology	Onset to hospital arrival	Onset to recanalization	Procedure time	Difficulty in vascular treatment	TICI	mRS at discharge
Cummings et al., 2012 [8]	100	N/A	3	10	9	-	Right M1	CES	N/A	390 min	40 min	No	3	4
Boo et al., 2015 [9]	103	F	0	30	10	+	Left ICA-T	CES	111 min	220 min	45 min	Artery tortuosity	3	1
Sweid et al., 2019 [10]	102	F	1	10	N/A	-	Right M2 with ICA stenosis	ATBI	170 min	276 min	62 min	Tandem lesion	3	1
	105	M	N/A	8	N/A	-	Left M2	CES	169 min	209 min	23 min	No	3	N/A Transfer to a hospice due to aspiration pneumonia.
Nguyen et al., 2020 [11]	103	F	0	30	10	+	Left ICA-T	CES	4 min	225 min	75 min	Artery tortuosity	3	1
Our case	100	F	1	24	10	-	Left M1	CES	31 min	129 min	37 min	No	3	2

Five of the six patients suffered a cardioembolic stroke, whereas one had an atherothrombotic brain infarction; however, arterial tortuosity rendered the management of two of the six patients difficult. Four of the six patients had good outcomes (mRS ≤2 at discharge). Preadmission mRS was 0-1 in all four cases, and good outcomes were reported even in cases with high NIHSS at the onset. The prognostic value of MT for LVO in centenarians remains difficult to predict, but activity level before onset appears to be important. It has been reported that in SAH, thickness and area of the temporal muscle correlate with the degree of sarcopenia, which may help predict prognosis after aneurysm intervention [13].

In reality, there is a publication bias, and it is believed that there are some cases with poor outcomes that have not been published; therefore, indications should be carefully judged. However, the fact that some centenarians are able to return home with good health after MT, as in our case, should encourage the use of MT for the very elderly.

## Conclusions

MT was successful in a centenarian with a left M1 occlusion. Typically, the greater the age of the elderly patient, the more stringent the indications for MT should be; however, no upper age limit is specified in the AHA/ASA guidelines. As we enter a super-aging society, the incidence of LVO in very old patients will increase. Further research on the efficacy and safety of MT for LVO in centenarians is needed.
